# Body Composition and Alzheimer’s Disease: A Holistic Review

**DOI:** 10.3390/ijms25179573

**Published:** 2024-09-04

**Authors:** Giulia Frank, Paola Gualtieri, Rossella Cianci, Mario Caldarelli, Roselisa Palma, Gemma Lou De Santis, Chiara Porfilio, Francesco Nicoletti, Giulia Bigioni, Laura Di Renzo

**Affiliations:** 1 PhD School of Applied Medical-Surgical Sciences, University of Tor Vergata, Via Montpellier 1, 00133 Rome, Italy; 2 School of Specialization in Food Science, University of Tor Vergata, Via Montpellier 1, 00133 Rome, Italy; 3 Section of Clinical Nutrition and Nutrigenomic, Department of Biomedicine and Prevention, University of Tor Vergata, Via Montpellier 1, 00133 Rome, Italy; 4 Department of Translational Medicine and Surgery, Catholic University of the Sacred Heart, 00168 Rome, Italy; 5 Fondazione Policlinico Universitario A. Gemelli, Istituto di Ricovero e Cura a Carattere Scientifico (IRCCS), 00168 Rome, Italy

**Keywords:** Alzheimer’s disease, body composition, obesity

## Abstract

Alzheimer’s disease (AD) represents a significant global health challenge and affects approximately 50 million people worldwide. This overview of published reviews provides a comprehensive understanding of the intricate correlations between AD and body composition, focusing particularly on obesity. We used a systematic approach to collect and analyze relevant reviews on the topic of obesity and Alzheimer’s disease. A comprehensive search of electronic databases, including PubMed, MEDLINE, and Google Scholar, was conducted. We searched keywords such as “Alzheimer’s disease”, “body composition”, “lean mass”, “bone mass”, and “fat mass”. We considered only reviews written within the past 5 years and in English. Fifty-six relevant reviews were identified that shed light on the multiple connections between AD and body composition. The review involves several aspects, including the impact of lean mass, bone mass, and endocrinological factors related to obesity, as well as inflammation, neuroinflammation, and molecular/genetic factors. The findings highlight the complex interplay of these elements in the development of AD, underscoring the need for holistic approaches to reduce the risk of AD and to explore innovative strategies for diagnosis, prevention, and treatment.

## 1. Introduction

Alzheimer’s disease (AD) is a neurodegenerative disorder that affects about 50 million people worldwide [1]. Previously, AD was diagnosed at the dementia stage, when significant and progressive cognitive decline has occurred and there is an evident impact on daily functions [2]. Therriault et al. conducted a prospective study using amyloid positron emission tomography (PET) and tau PET to evaluate the prevalence of biologically defined AD in relation to age, sex, serum markers, and clinical diagnosis [3]. They concluded that the clinical diagnosis of probable AD shows an agreement with biomarker positivity [3] but underestimates the real frequency of diagnosis of AD.

AD is characterized by the progressive loss of neurons and synapses in the cerebral cortex and some subcortical regions due to the accumulation of β-amyloid (βA) protein that is abnormally folded into amyloid plaques. The diagnosis and monitoring of and the evaluation of treatment efficacy for AD rely significantly on biomarkers, which can be classified into three types [4]: (a) neurodegenerative markers, including those detected by structural/functional imaging and levels of tau and neurofilament light in the cerebrospinal fluid (CSF); (b) biomarkers of tau, such as the detection of p-tau181 in CSF and p-tau217 in plasma or through PET imaging of tau; (c) and biomarkers of βA, such as those detected via PET imaging of amyloid and βA42 levels and the βA42/βA40 ratio in the CSF [5,6].

The pathogenesis and causes of the neurodegenerative progression of AD are multifactorial, and risk factors are multifaceted. About 5% of cases have familial ties that are associated with mutations in the presenilin-1 (*PSEN1*) or *PSEN2* genes and amyloid protein precursor (*APP*), while late-onset AD lacks identified genetic variants [7,8]. Key AD risk genes have been recognized, including apolipoprotein E (*APOE*) and triggering receptor expressed on myeloid cells 2 (*TREM2*), which influence the complement system. *APOE* isoforms modulate complement activation, thereby affecting synaptic pruning and integrity [9]. The role of *TREM2* in complement-mediated synaptic pruning is complex, with conflicting evidence suggesting a dual effect in the context of AD [10]. The impact of sexual dimorphism in complement signaling is evident, particularly in the increased expression of the complement protein C1q in aging female brains, shedding light on the greater susceptibility of women to AD [11].

Various environmental risk factors may contribute to AD onset.

The “dual hit” model suggests that environmental stressors activate genes in early development, modifying gene expression later in life and ultimately contributing to AD onset. Another theory, the “allostatic load”, suggests that stress accelerates premature aging, resulting in neuronal degeneration, synapse disconnection, and the activation of genes producing βA and tau proteins, which are all implicated in AD pathology [7].

Lifestyle factors, such as sleep patterns, exercise, diet, and exposure to pollutants and metals significantly influence the AD risk.

Cardiovascular risk factors also contribute to the pathogenesis of AD.

Gui et al. aimed to examine the associations of cardiovascular risk factors (e.g., current smoking and alcohol consumption, physical inactivity, obesity, total cholesterol, triglycerides, low-density lipoprotein cholesterol (LDL-c), high-density lipoprotein cholesterol, diabetes, and hypertension) and variants of *APOE* and translocase of outer mitochondrial membrane 40 (*TOMM40*) with global cognitive function through the use of the Mini Mental State Examination (MMSE) score in elderly individuals [12]. Both the *APOE* gene and several single-nucleotide polymorphisms of *TOMM40* are well-known risk factors for the development of AD [13,14].

The findings indicated that some *TOMM40* polymorphisms, regardless of the *APOE ε4* allele, interacted with combined cardiovascular risk factors (*VRFs*) to affect cognitive performance. Possessing one or more of these *VRFs* was particularly harmful to the cognition of *TOMM40* carriers. Further analysis showed that the *TOMM40* polymorphism interacts with physical inactivity and diabetes. Low physical inactivity or diabetes along with a *TOMM40 G* allele are significantly linked to a notably lower MMSE score [12].

Nianogo et al., in a cross-sectional study, investigated whether modifiable risk factors (such as obesity, physical inactivity, and low education) associated with AD have changed in the United States over the past decade and whether these factors differ by sex, race, and ethnicity [15]. The results indicate that these modifiable risk factors that are linked to AD have evolved over the last ten years and that they vary by sex and race/ethnicity; AD cases linked to modifiable risk factors were more numerous among men and among American Indian, Alaska Native, Black, and Hispanic individuals (of any race) than among Asian and White individuals [15].

Epigenetic factors, particularly DNA methylation, histone modifications, and miRNA regulation, play crucial roles in AD, impacting the susceptibility to oxidative DNA damage, and are associated with AD-susceptible genes. DNA methylation has been shown to be a key regulatory mechanism in AD, along with contributions from microRNA-126 and mitochondrial DNA variations [16].

The MEMENTO study aimed to demonstrate the usefulness of plasma and cerebrospinal markers in clinical practice [17].

The MEMENTO study included 2323 outpatients experiencing subjective cognitive complaints or mild cognitive impairment. At the start of the study, participants underwent neuropsychological evaluations, MRI scans, and blood tests. Baseline measurements of the blood βA42/40 ratio and the levels of total tau, p181-tau, and neurofilament light chain (NfL) were conducted using a Simoa HD-X analyzer [17].

Higher concentrations of p181-tau and NfL in blood and CSF were linked to a faster onset of AD dementia, with similar rates of incidence. However, the blood βA42/40 ratio was found to be less effective than the CSF βA42/40 ratio as a prognostic marker [17].

Moreover, when cerebral pathology was defined using amyloid PET, the blood βA42/40 ratio decreased by 16% in βA+ participants. Among the blood biomarkers, p181-tau showed the strongest association with cerebral amyloidosis [17].

βA is the main target of the most recent pharmacological therapies using passive immunotherapy.

A recent phase III clinical study by Sperling et al. reported that solanezumab, a monoclonal antibody targeting the βA peptide, failed to slow cognitive decline in AD patients [18].

Other anti-βA antibodies, such as lecanemab (a humanized mAb that binds to soluble βA protofibrils), donanemab (a humanized mAb that binds to the insoluble, N-terminal truncated form of βA peptides), and aducanumab (a human mAb that binds to the aggregated form of βA), have shown success in slowing cognitive decline in early-stage AD patients [19].

γ-Secretase, a complex protein, has emerged as another therapeutic target, with modulators demonstrating the potential for intervention. Understanding the mechanisms influencing the βA42/βA40 ratio is vital for comprehending AD’s origin, and γ-secretase plays a pivotal role in regulating the βA protein length and ratios. Over 300 mutations in *PSEN*-encoding genes impact γ-secretase function, and therapeutic approaches using γ-secretase modulators hold promise for reducing βA42 levels without adverse effects, reinforcing γ-secretase’s significance as a genetically and biochemically linked factor in AD with therapeutic potential [20].

Obesity is increasingly being recognized as a potential AD risk factor, with inflammation playing a pivotal role in connecting these two conditions. Beyond the overall body weight, body composition, particularly the distribution of adipose tissue [21] and sarcopenia [22], contributes significantly. Excessive adiposity, especially visceral fat, is associated with the release of proinflammatory cytokines, fostering a chronic state of systemic inflammation. This inflammatory environment is implicated in neuroinflammation, a key contributor to AD pathology [23]. Understanding the interplay between obesity, inflammation, and body composition is crucial for developing targeted interventions that address both cognitive health and the broader impact of adipose tissue distribution on neurodegenerative processes in AD.

Thus, the purpose of this overview of reviews [24] is to provide an update on the latest scientific evidence in order to understand the possible correlations between AD and body composition. In particular, the role of excess fat mass in AD will be clarified from an endocrinological, immunological, and genetic perspective.

## 2. Results

From the research conducted, 11 reviews were focused on the role of lean mass [25,26,27,28,29,30,31,32,33,34,35], 5 reviews focused on bone mass [36,37,38,39,40], and the remaining reviews focused on fat mass. Regarding the latter, we found 19 articles dealing with the topic from an endocrinological point of view [41,42,43,44,45,46,47,48,49,50,51,52,53,54,55,56,57,58,59], 14 articles from an immunological point of view [60,61,62,63,64,65,66,67,68,69,70,71,72,73], and 7 articles from a molecular and genetic point of view [74,75,76,77,78,79,80].

The characteristics of all the articles on lean mass and AD are listed in Table 1.

The characteristics of all the articles on bone mass and AD are listed in Table 2.

The characteristics of all the articles on fat mass and AD from an endocrinological perspective are listed in Table 3.

The characteristics of all the articles on fat mass and AD from an immunological perspective are listed in Table 4.

The characteristics of all the articles on fat mass and AD from a molecular and genetic perspective are listed in Table 5.

## 3. Discussion

### 3.1. Body Composition and Main Findings in Alzheimer’s Disease

AD development is often preceded by weight loss. While a higher body mass index (BMI) in middle age has been consistently associated with cognitive impairment, the nonlinear relationship between the BMI and dementia risk in later life suggests that the BMI may not adequately measure adiposity, especially in relation to regional fat distribution and muscle mass [81]. In particular, Larsonn et al. [82] reported that although BMI is associated with several diseases, it is not associated with AD. Thus, it is critical to investigate the associations between regional fat, skeletal muscle mass loss and function, the brain, and cognition in order to clarify the role of body composition in AD.

### 3.2. Lean Mass and Main Findings in Alzheimer’s Disease

The role of lean body mass (LBM) in AD and its potential modulation through exercise and physical activity have been extensively discussed by various researchers.

Wu et al. [25] underscored the connection between early-stage AD and sarcopenia, which was influenced by factors such as genetics, lifestyle, and inflammation. They highlighted the bidirectional relationship between cognitive impairment and sarcopenia, indicating shared risk factors and genetic elements. Additionally, animal models demonstrated AD-related muscle deficits, with the APPswe gene expression in muscles being linked to brain pathology and age-dependent behavior abnormalities, thereby suggesting potential therapeutic avenues for AD.

Furthermore, Correa-de-Araujo et al. [26] reported age-related changes in body fat distribution, with emerging evidence suggesting increased intramuscular adipose tissue, which particularly varied by sex and race. They stressed the need for comprehensive, multiethnic, and longer-term studies to unravel the intricate interplay between myosteatosis and aging, including in AD, taking into account factors like sex, race, ethnicity, and weight history.

Ingenbleek et al. [27] highlighted the impact of inflammatory disorders on LBM, indicating that cytokine-induced tissue breakdown affects LBM, which typically encompasses most nitrogen-containing molecules. This breakdown alters the sulfur-to-nitrogen ratio in healthy tissues, reflecting changes in hepatic transthyretin (TTR) secretion, which normally exerts neuroprotective activities. Thereby, malnutrition, characterized by gradual LBM downsizing and reductions in body stores of essential amino acids like methionine, can impede LBM protein synthesis and accretion, diminishing the TTR neuroprotective activities.

Suryadevara et al. [28] discussed the association between AD progression and skeletal muscle mass, strength, and function. They suggested that muscle dysfunction might precede neurological symptoms and serve as a predictor of cognitive decline. Skeletal muscle’s role in insulin activity and glucose regulation was highlighted, offering a potential therapeutic target for AD. Reduced LBM and strength were linked to AD severity, increasing the risk of falls and fractures, while poor gait was considered a prodromal sign of cognitive decline. Muscle function defects, particularly dynapenia, were proposed as early predictors of AD progression.

Additionally, Halon-Golabek et al. [29] emphasized the association between iron accumulation in skeletal muscle, oxidative stress, and impaired insulin signaling, drawing parallels with the similar dysregulation observed in neurodegenerative diseases like AD. Indeed, dysregulation of insulin signaling, mediated by the Akt/FOXO3a pathway, can disrupt iron metabolism, contributing to iron accumulation and oxidative stress in skeletal muscle. They also highlighted the role of myokines, especially irisin, in AD pathology, noting its decline in AD-affected brain regions and its potential neuroprotective effects. Moreover, apelin, another myokine, has been implicated in neuroprotection and in the inhibition of amyloid beta production in the brain.

Han et al. [30] explored the intricate relationship between sarcopenia, cognitive impairment, and AD, suggesting a strong association between them. They identified beneficial factors like insulin-like growth factor-1 (IGF-1), brain-derived neurotrophic factor (BDNF), irisin, and secreted protein acidic and rich in cysteine (SPARC), which play roles in maintaining muscle and cognitive function. Conversely, detrimental factors such as interleukin-15 (IL-15) and myostatin were also discussed. They proposed aerobic and resistance exercises as promising interventions due to their ability to maintain muscle mass and cognitive health.

Several other articles have highlighted the benefits of physical exercise on LBM in AD [29]. Particularly, Brisendine et al. [31] highlighted the significant loss of lean muscle mass and strength in the preclinical phase of AD, emphasizing the association between skeletal muscle mass, mitochondrial function, and cognition. While evidence for the effectiveness of exercise in AD treatment is conflicting, recent studies suggest potential benefits, particularly with resistance exercise, in preserving cognitive function and reducing AD-related pathology.

Similarly, García-Llorente et al. [32] conducted a meta-analysis focusing on multidomain exercises, revealing varying outcomes regarding cognition improvement and muscle strength, which are likely due to differences in training parameters. However, functional strength showed improvement in some studies, suggesting the effectiveness of multidomain exercises in reducing the risk of falls. The number of weekly exercise sessions influenced the results, highlighting the importance of considering different exercise parameters for their effective prescription in elderly populations.

Jodeiri Farshbaf et al. [33] emphasized the role of fibronectin type III domain-containing protein 5 (*FNDC5*)/irisin expression in AD. While exercise boosts *FNDC5* expression in the hippocampus and prefrontal cortex, decreased *FNDC5* expression is observed in AD-affected brain regions. Irisin levels are low in individuals with post-stroke depression, yet irisin administration has shown promise in alleviating depression, stress-induced anxiety, and memory impairment. The regulation of *FNDC5*/irisin involves various signaling pathways, including PGC-1α and cAMP/PKA pathways, that are influenced by physiological conditions like exercise and environmental enrichment. Irisin has been implicated in reducing βA deposits, reducing apoptosis and inflammation in neuronal cells, and enhancing memory and cognition through synaptic plasticity and neurogenesis. Furthermore, sex hormones, particularly estrogen (E2), also play a role in AD pathology. E2 influences *FNDC5*/irisin expression in skeletal muscle and impacts neuroprotective pathways in the brain, with implications for sex-specific effects on memory.

Chen et al. [34] correlated irisin plasma levels ranging from 3.6 to 4.3 ng/mL with BDNF and βA levels, highlighting its role in promoting learning and memory through various pathways, including cAMP→PKA→CREB; moreover, irisin induces BDNF production directly in the brain or via downstream signaling cascades. Irisin also exerts anti-inflammatory effects by reducing the release of inflammatory cytokines and inhibiting the NF-κB pathway, thus mitigating neuroinflammation in AD. It modulates astrocytes to protect neurons from βA toxicity and promotes neuronal cell viability through various pathways, including the reduction of IL-6 and IL-1β release and the inhibition of *COX-2* expression. Additionally, irisin improves insulin resistance and glucose homeostasis via the PI3K/Akt and p38MAPK signaling pathways, further emphasizing its potential as a therapeutic target for AD.

Furthermore, Cao et al. [35] emphasized the significant impact of exercise on AD risk factors, particularly skeletal muscle, liver, and adipose tissue metabolism responses. While exercise offers protection against AD, further research is needed to fully understand its mechanisms.

In conclusion, lean body mass has emerged as a crucial factor in AD, with exercise remaining a potent intervention for modifying the effects of aging on metabolism and health. Irisin, in particular, holds promise as a therapeutic target for AD, offering multifaceted benefits through its actions on the brain and peripheral tissues. However, many of these studies did not take into account confounding factors, such as sex and education. Further research is warranted to elucidate its precise mechanisms and therapeutic potential in AD treatment.

### 3.3. Bone Mass and Main Findings in Alzheimer’s Disease

Karnik et al. [36] showed that AD and osteoporosis have the same few risk factors, including older age, female gender, genetic susceptibility, and lifestyle factors. Furthermore, molecular features typical of AD, including oxidative stress, chronic inflammation, and the accumulation of the βA and tau proteins, are linked with a dysregulation of bone metabolism and an increased risk of fracture. On the other hand, impaired bone health may promote the progression of AD. In the US, women account for approximately two-thirds of those affected by AD, and it has been shown that this is due not only to their greater longevity but also to a multifactorial hormonal and genetic predisposition. Aging is associated with chronic low-grade inflammation, which would seem to underly the pathologies of both AD and bone disease.

Suryadevara et al. [28] showed that AD correlates with decreased bone mass density (BMD), and the incidence of fractures increases as the AD progresses. Women with osteoporotic fractures have an increased risk of developing AD. The use of psychotropic medications is associated with an increased risk of hip fracture. In one study, the prevalence of AD was higher in postmenopausal women with severe osteoporosis, especially after proximal femur fractures. Women with AD have lower BMD than men. Reduced BMD correlates with the amount of brain atrophy and a decline in cognitive performance. Tg2576 mice, a preclinical model of AD, have reduced bone formation compared with wild-type mice.

Recent evidence highlights a bidirectional cause–effect role between AD and bone pathology. In particular, the neuroinflammation present in AD seems to cause dysregulation of the hypothalamic–pituitary–adrenal axis with a consequent increase in cortisol, which would consequently lead to a loss of bone density. Conversely, chronic inflammation resulting from osteoporosis and fractures increases neuroinflammation and could contribute to the pathogenesis of AD. Patients with AD have an increased risk of fractures due to the increased incidence of falls and the reduced BMD associated with reduced mobility and nutrition, which is typically seen in AD patients. On the other hand, although it seems that fractures might promote the progression of AD, there is currently insufficient evidence to support this.

Frame et al. [37] proposed that AD may begin its degenerative process in evolutionarily conserved central nervous system (CNS) regions governing basic homeostatic functions, impacting skeletal remodeling pathways. Dysfunction in CNS pathways, specifically in the dorsal raphe nucleus and ventromedial hypothalamus, has been identified as an early site of pathology in AD, potentially contributing to bone remodeling abnormalities. Despite not affecting every individual with AD, clinically significant bone loss occurs in more than twice as many AD patients compared with the general age-matched population. This study shows that skeletal impairments develop very early in the clinical course of AD and cannot be attributed to other factors, such as aging, gender, mobility, falls, or genetics. Although research in this area is still in its early stages, studies suggest several potential mechanisms that may disrupt the skeletal homeostasis, including direct effects of amyloid-beta on bone cells, tau-induced damage to neuronal centers involved in bone remodeling, and/or defects in systemic Wnt/beta-catenin signaling. Increasing evidence also points to the role of the recently identified “exercise hormone” irisin and its precursor protein FNDC5 in bone loss and neurodegeneration [37].

Additionally, Zhou et al. [38] highlighted that patients with AD have an increased risk of fractures; in particular, hip and vertebral fractures. AD patients often have reduced BMD compared with non-AD patients. The mechanisms that increase the risk of fractures in AD patients are multifactorial. The βA peptide may directly interfere with skeletal remodeling by affecting bone cells. A previous study found that βA can influence osteoclast differentiation both in vitro and in vivo. Additionally, emerging evidence suggests that advanced glycation end products (AGEs) play a role in the development of Alzheimer’s disease and may act as a seed for βA aggregation. The receptor for AGEs (RAGE), which binds to βA, has also been implicated in the AD pathogenesis [38]. Similarly, neuronal tissue influences skeletal tissue.

The sympathetic nervous system (SNS) and parasympathetic nervous system (PNS) can affect bone through various pathways involving circadian genes; the neuropeptide Y, serotonin, leptin, adiponectin, muscarinic, nicotinic, and beta-adrenergic receptors; and the sensory innervation of bone [38]. Moreover, AD patients typically show increased sympathetic activity and decreased parasympathetic and cholinergic innervation, especially in the elderly. Indeed, AD patients treated with AChE inhibitors show a reduced risk of hip fracture and improved bone healing, suggesting that impaired parasympathetic signaling affects bone homeostasis and may be a potential target for improving bone health in these patients.

In addition, the Wnt/β-catenin pathway influences both osteoblast differentiation and bone formation, while in the brain, it also promotes the development of synaptic connections between neurons and enhances neuronal survival [38].

Lastly, neuroinflammation plays a key role. Chronic neuroinflammation and the over-activation of resident microglial cells, the infiltration of macrophages, and the involvement of circulating immune cells may contribute to the AD pathogenesis. Levels of tumor necrosis factor-alpha (TNF-α) in the cerebrospinal fluid (CSF) and serum of AD patients were significantly elevated compared with healthy controls and were associated with disease progression. TNF-α directly enhances bone remodeling (partly through synergistic interactions with RANKL) by promoting osteoclast differentiation and, consequently, bone resorption [38].

Ruggiero et al. [39] revealed a bidirectional relationship between cognitive impairment, dementia, and fragility fractures. Early cognitive impairment leads to impaired mobility, which, in turn, leads to reduced BMD and an increased risk of falls and fractures. In particular, patients with AD have twice the risk of fragility fractures compared with people without AD. Older adults with dementia or cognitive impairment have an increased risk of fragility fractures and vice versa. In a 12-year retrospective analysis, individuals with a history of fractures showed a 41% increased incidence of dementia. Individuals aged >65 years have a 60% risk of dementia following hip fractures, a 47% risk following vertebral fractures, and a 35% risk following lower limb fractures. Furthermore, low BMD could be used as an early marker of later cognitive impairment. Subjects with a low BMD have an increased risk of dementia and, particularly, AD compared with subjects with a normal BMD. It seems that treatments with bisphosphonates or estrogen would mitigate the development of dementia.

Zhao et al. [40] highlighted that AD and osteoporosis have common risks, such as the risk of falling, loss of muscle function, impaired balance and gait, and death. Osteoporosis treatment could prevent or delay the onset of cognitive impairment. Specifically, AD is associated with βA deposition, and it was highlighted that βA can increase RANKL activation, and thus osteoclast activation, by increasing bone resorption. In addition, low levels of osteocalcin, a marker of bone formation, reflect both the severity of osteoporosis and of cognitive impairment. Some evidence shows that cognitive impairment and osteoporosis have common risk factors: age, depression, reduced social activities, reduced sex hormones and calcium levels, activated vitamin D levels, and cytokine alterations. Estrogens maintain bone health, and a meta-analysis showed that estrogen replacement therapy can improve cognitive function in patients with AD.

Given the fundamental role of inflammation in the pathogenesis and progression of AD and bone disease, the investigation of therapies that counteract the inflammatory pathways common to both pathologies should be encouraged.

### 3.4. Fat Mass and Main Findings in Alzheimer’s Disease from an Endocrinological Perspective

An excess of fat mass is an important risk factor for AD development and progression [45], and several studies have analyzed the mechanisms involved in the interaction between obesity and AD [41,42,43,45,48,49,50,51,52,53,54,55,57,58,59].

Neto et al. [45] observed how an obesity-related energy imbalance contributes to synaptic loss and memory issues in AD mouse models [45]. Several studies indicate a connection between being overweight or obese and brain volume loss, particularly in regions affected by AD pathology, such as the hippocampus [45]. Across adulthood, a higher BMI is associated with reduced brain perfusion, reflecting a negative impact of obesity on brain function [45]. Moreover, a higher BMI and waist circumference (WC) are linked to reduced cortical thickness, suggesting a global impact of obesity on the brain. Additionally, an increased WC is associated with a decrease in gray matter volume [45]. Obesity-related metabolic changes can also trigger the development of harmful substances called AGEs, which promote βA clustering and damage to the CNS [45].

Huang et al. [58] suggest a bidirectional relationship between the brain and visceral white adipose tissue (vWAT) and its contributions to AD development and progression.

In addition, excess fat mass is related to several comorbidities, such as insulin resistance, diabetes mellitus, and metabolic syndrome. In particular, Abdalla [46] highlighted a possible link between diabetes and an increased risk of developing AD, which is associated with the protective role of insulin in brain function. In fact, a dysfunction in insulin signaling, such as in conditions of insulin resistance or diabetes, results in the accumulation of βA plaques and tau protein tangles, which are characteristic of AD.

Moreover, Arjunan et al. [47] reported how insulin growth factor-1 (IGF-1) deficiency, which is evident in metabolic syndrome, results in molecular signaling defects that occur in neurodegenerative disorders such as AD. Indeed, IGF-1 plays a key role in protein clearance in the brain, angiogenesis, neurotropism, metabolic function, neuronal plasticity, and embryonic and adult neurogenesis.

Fat has not only an energy reserve function, but it also has endocrine activity, secreting several hormones called adipokines [41,45,48,50,54]. Adipokines modulate low-grade inflammation, which is found in some diseases, including obesity and AD [42,45,48,50,54]. Leptin and adiponectin are the two major protein hormone secreted by white adipose tissue, that have a preventive role against obesity and dementia, and both are involved in the causal relationship between obesity and cognitive decline [45,48,50].

Several reviews examine the relationship between leptin and AD [42,44,45,48,50,51]. Leptin regulates the homeostasis of food intake [42,44,45] and energy expenditure [42,45], induces satiety [48,50,51], and increases insulin sensitivity and lipolysis [48]. It has a neuroprotective action [42,44,45,48] and reduces the risk of developing cognitive decline [48] and AD [45] as it reduces βA plaque formation [45]. Leptin also mitigates the negative effects of βA on synaptic function and memory deficits [45]. Its secretion increases in obesity and in cases of increased food consumption [42,50,51]. However, chronic obesity-related low-grade inflammation causes leptin resistance, which, in turn, increases the risk of cognitive decline [42,48] and AD [42,45,50]. Leptin resistance causes impaired satiety, causing a vicious cycle [42,50].

It has been demonstrated that the amount of leptin increases during aging because of increased adipose tissue. However, during aging, leptin resistance occurs [51].

Khoramipour et al. [52] highlighted that adiponectin is an adipokine with several beneficial effects on different organs [52,54,55,57]. It increases insulin sensitivity; reduces oxidative stress, LDL-c levels, and inflammation; activates the immune system; acts as an anti-atherogenic agent; protects against diabetes; and has neuroprotective effects [44,51,52,54,55,57]. Moreover adiponectin manages energy expenditure, carbohydrate metabolism, and fatty acid catabolism, preventing obesity [41,48,51,55]. In the brain, it promotes hippocampal neurogenesis and neuronal plasticity, reduces oxidative stress [48], counteracts βA plaque formation [45], and has positive effects on cognitive function [48,52]. Its secretion increases following a healthy diet and weight loss [48,52]. Low levels of adiponectin are found in obese individuals and promote the onset of neurodegenerative diseases and cognitive decline and the onset and progression of AD [48,51,52,54,55,57]. In addition, low levels of adiponectin are also found in AD subjects [45,55], suggesting a causal role of obesity in promoting the onset of AD [54,55,57]. Therefore, obesity prevention would reduce the risk of AD [55]. In addition, Kim et al. [55] also attribute a causal role for adiponectin in the onset of AD from type 2 diabetes. Adiponectin can be considered a metabolic marker of AD [55].

Sindzigre et al. analyzed the role of adiponectin in AD patients in preclinical and human studies [41]. Preclinical studies show that adiponectin has a neuroprotective role in preventing AD [41]. Indeed, a reduction in adiponectin or the suppression of its receptor, AdipoR1, leads to neurodegeneration and the deposition of βA plaques [44]. However, from the analysis performed by Sindzigre et al. [41], the human studies showed conflicting results.

In a recent review, Forny-Germano et al. [56] supported the involvement of leptin and adiponectin as critical mediators of obesity-related CNS dysfunction. This review found that leptin and adiponectin, together with their receptors, are present in the brain and play important roles in regulating brain physiology. In particular, leptin and adiponectin signaling has been shown to influence several neuropathological processes that are common in neurodegenerative diseases, particularly in AD. These processes include amyloidogenesis, tau hyperphosphorylation, neuroinflammation, oxidative stress, endoplasmic reticulum stress, insulin resistance, synaptic dysfunction and cognitive impairment. Remarkably, mice deficient in adiponectin or its receptors show most of the neuropathological features characteristic of AD. Thus, impaired adiponectin and leptin signaling may contribute to the negative effects of obesity on the CNS and increase the risk of cognitive decline and AD. Restoring proper leptin and adiponectin signaling in the brain could potentially offer beneficial disease-modifying therapeutic approaches for these neurological conditions.

In addition, Kim et al. [55] draws attention to the effects of pharmacological treatments for AD and type 2 diabetes. In particular, some studies would seem to show that AD drugs result in an increase in adiponectin and vice versa; thus, it would seem that pharmacological treatments of type 2 diabetes could have a positive effect on AD.

Uddin et al. [57] suggest that further studies should investigate the possibility of using adiponectin as a treatment to reduce the progression of AD.

Huber et al. [44] examined the involvement of thirteen adipokines in CNS pathologies. Specific adipokines play a promoting, inhibitory, or modulatory role in the genesis of different pathologies. In addition to the already mentioned leptin and adiponectin, other adipokines examined in this review are cystatin C and chitinase 3-like protein 1 (CHI3L1). Cystatin C has a neuroprotective role and low levels of cystatin C in the CSF correlate with rapid dementia progression, suggesting an association with AD [44]. CHI3L1 disrupts tight-junction expression in the blood–brain barrier (BBB), is involved in βA accumulation, and promotes inflammation, which ultimately promotes AD. Increased levels of CHI3L1 could potentially serve as biomarkers for patients in the preclinical and early stages of AD [44].

Several studies have investigated the relationship between insulin and AD in obese patients [43,45,51]. Insulin resistance is a feature of obesity, metabolic syndrome, and type 2 diabetes, and it is linked with cognitive impairment [49]. Increased insulin resistance correlates with higher amyloid deposition in middle-aged humans [45]. Mechanisms linking brain insulin resistance and AD include hyperinsulinemia; the competition between insulin-degrading enzymes and βA; the binding of βA to insulin receptors, impairing signaling pathways; and the downregulation of insulin receptors due to βA binding [45].

Andrade et al. [43] have highlighted that central insulin resistance is present in several neurodegenerative diseases, such as AD. Although some studies show that impaired brain glucose utilization and uptake results in reduced ATP production, which correlates with reduced neuronal plasticity and cognitive impairment, some studies do not support a relationship between central insulin resistance and AD [43]. Therefore, further studies are needed to determine whether or not there is indeed a causal relationship [43].

Cimini et al. [49] analyzed one of the mechanisms promoting the development of insulin resistance. Recent studies have highlighted that biliverdin reductase-A (BVR-A), in addition to its well-known role in the degradation of heme, acts as a regulator of insulin signaling. Insulin signaling in the brain plays a pivotal role in the regulation of hippocampal plasticity as well as in learning and memory functions. Evidence shows that reduced BVR-A levels or impaired BVR-A activation contributes to the development of brain insulin resistance and metabolic alterations encountered in AD-affected patients. The experimental data suggest that therapies targeted at restoring metabolic homeostasis may improve cognitive function and increase the lifespan in neurodegenerative diseases. Likewise, uncontrolled, progressive weight gain and abnormal glucose tolerance are common metabolic dysfunctions observed in AD, which appear to negatively impact the overall prognosis. AD could be regarded as a metabolic disease mediated in part by brain insulin resistance.

AD is associated with age and is characterized by inflammation, called inflammaging, and insulin resistance. Komleva et al. [53], in their review, analyzed the role of nutrients, obesity, obesity-linked low-grade chronic inflammation in brain aging, neurodegeneration, and cognitive decline. In addition, their review considered how calorie restriction and lifestyle changes could break this vicious cycle, leading to healthy aging and slowing the onset of neurodegenerative diseases.

Farruggia et al. [59] analyze the role of obesity and metabolic dysfunction in the pathogenesis of dementia. In particular, this review highlighted that in animal models, adiposity and metabolic dysfunction, such as type 2 diabetes mellitus and insulin resistance, independently affect cognitive abilities. However, in humans, since obesity and metabolic dysfunction often coexist, it has not been possible to clarify whether obesity and metabolic dysfunction are independent factors in the development of cognitive dysfunction. The overall relative risk of AD is 46% in those with type 2 diabetes compared with those without. AD and type 2 diabetes share some pathogenetic mechanisms, including inflammation, insulin resistance, and mitochondrial dysfunction.

Chung [51] shows that during aging, the amount of adipose tissue increases due to an increased uptake of fatty acids at the cellular level and the failure to oxidize them. Therefore, ectopic fat formation occurs, which is responsible for lipotoxicity, reduced energy availability, and altered cellular signaling mechanisms. These factors underlie the development of aging-related diseases, including AD.

### 3.5. Fat Mass and Main Findings in Alzheimer’s Disease from an Immunological Perspective

In the last few decades, several studies have focused on the role of the immune system in AD onset and progression. Both systemic inflammation and neuroinflammation are involved in AD [60,61].

Obesity has emerged as an independent risk factor for AD, with high-fat diets linked to hyperinsulinemia and insulin resistance, which are other known risk factors for AD [60,61]. Although the exact mechanisms linking obesity to AD have not been fully elucidated, systemic inflammation and subsequent neuroinflammation appear to play significant roles. Neuroinflammation, in particular, results in the activation of microglia cells, which are the immune system cells residing in the brain [61].

In AD, the loss of BBB integrity allows immune system cells to migrate into the CNS, exacerbating neurodegeneration and inflammation. Obesity-related inflammation further increases BBB permeability. Neuroinflammation induces the efflux of CNS proteins, including βA and inflammation mediators, across the BBB, which can trigger a systemic immune response and recruit myeloid and lymphocytic cells in the CNS [62].

Cytokines secreted by adipose tissue, such as IL-1β, IL-10, and IL-18, are elevated in both obesity and AD. Obesity-related dysregulated cytokine production in adipose tissue may contribute to AD development and progression. The role of autophagy in counteracting AD and the involvement of adipose-derived molecules in autophagy processes also warrant further investigation [63].

Some studies have highlighted how prebiotics can reduce AD symptoms by improving good microbiota and probiotics by reinforcing the gut barrier function [64]; on the other hand, an increase of dietary and metabolic factors that can elicit a higher response of Th17 cells and their cytokines (IL-17, IL-22) may determine cognitive impairment, even in the absence of amyloid plaques [65].

The potential biological mechanisms underlying the connection between obesity and both neurodegeneration and neurodevelopment have been extensively examined [66]. Obesity results from an excessive intake of energy, leading to the hypertrophy of adipose tissue. Malfunction and inflammation within the adipose tissue initiate compromised insulin signaling, the altered secretion of adipokines and cytokines, impaired triglyceride storage, and the release of free fatty acids. Increased levels of free fatty acids are linked to insulin resistance (IR), which has been correlated with neurocognitive dysfunction. IR and sustained hyperglycemia provoke oxidative stress and inflammatory responses, leading to neuronal death and impairing cognitive functions [67].

Recent studies have linked obesity to neuroinflammation through BBB impairment. In mice fed with a high-fat diet, activation of microglia and of Th1 and Th17 cells has been shown; on the contrary, the use of short-chain fatty acids, such as propionate, can increase the amount of Tregs [45]. Visceral adipose tissue (VAT) has been shown to play a role in systemic inflammation. The most studied mediators of adipose tissue are represented by adiponectin and leptin. While adiponectin shows an anti-inflammatory effect by inhibiting *IL-6*, *TNF-α*, *TLR2*, *TLR5*, and *TLR7* expression and the nuclear factor kappa-light-chain-enhancer (*NF-kB*), and it is lowered by systemic inflammation, leptin can enhance Th1 cells and macrophage activity [68]. Moreover, adipose tissue generates inflammatory cytokines that are closely associated with the development of neurodegenerative diseases [69]. These cytokines are IL-1β, IL-4, IL-10, IL-18, and TNF-α [63]. The deposition of tau protein is enhanced by the resistance to leptin [70]. Furthermore, in obesity, NLRP3 inflammasome can elicit chronic inflammation.

In obese people, a trend of protein deficiency has also been reported. The assessment of plasma TTR has been suggested as a responsive indicator of the protein nutritional status [71]. Schwarzman et al. demonstrated that TTR could impede the formation of βA amyloid84, a focal point in numerous current research studies [72]. TTR revealed the strongest positive association with LBM (r = 0.64), while retinol-binding protein (RBP) showed a moderate correlation (r = 0.57), and albumin exhibited the lowest correlation (r = 0.52) [73]. In protein-depleted conditions, limiting both dietary protein and methionine (Met) downregulates the hepatic production of TTR [27]. TTR values also demonstrate a distinctive ability to incorporate the extent of nitrogen (N) depletion, whether stemming from incomplete LBM replenishment during protein malnutrition or from substantial LBM losses induced by cytokine-driven inflammatory stress [27]. The synergy between protein malnutrition and the consequent release of inflammatory cytokines heightens the susceptibility to irreversible neurodegenerative consequences [27].

### 3.6. Fat Mass and Main Findings in Alzheimer’s Disease from a Molecular and Genetic Perspective

Genetics plays a significant role in the development of obesity and neurodegenerative diseases. A central example is that of *APOE*, a glycoprotein primarily produced by astrocytes in the brain and peripherally in the liver [67].

Zhao et al. conducted a study to examine the relationship between obesity and AD; they showed that *APOE* was the most frequently occurring target [83].

In the AD population, the distribution of alleles of the *APOE* gene is as follows: *ε3* comprised the majority of the *APOE* gene pool at 58%, while *ε2* and *ε4* accounted for 4% and 38%, respectively [84].

*APOE* is present in various forms in both the peripheral circulation and CNS [85].

In the peripheral circulation, *APOE* plays a role in the redistribution and metabolism of triglycerides, cholesterol, cholesterol esters, phospholipids, and other lipids by forming lipoprotein particles, thus maintaining lipid homeostasis [86].

*APOE* in the peripheral circulation can regulate brain function either by directly affecting the endothelial cells of the BBB or by indirectly influencing endothelial and neuronal functions through lipid metabolism, atherosclerosis, and peripheral inflammation [83]. 

One possible hypothesis is that as the BMI, body fat percentage (BFP), and total fat percentage (TFP) increase, the expression level of *APOE3* decreases, and this reduction could potentially lead to the development of AD [83].

Moreover, The Framingham Heart Study explored the links between obesity and the expression of genes associated with AD in a sample of 5619 participants. Gene expression was analyzed for a panel of 74 AD-related genes, identified by combining genome-wide association study findings with functional genomics information [87].

The study found that obesity was linked to the expression of 21 genes related to AD. The most significant associations were seen with *CLU*, *CD2AP*, *KLC3*, and *FCER1G* [87].

Another instance of shared genetic factors between obesity and neurodegenerative diseases involves leptin and its receptor, *LEPRβ*. *LEPRβ* triggers the JAK-2/STAT3 pathway [74]. Recent studies have emphasized the importance of leptin and its receptor in neurological conditions and neurodevelopmental disorders. Laboratory research has shown that inhibiting the JAK/STAT3 pathway provides a defense against the neuroinflammation caused by α-synuclein (α-SYN) and dopaminergic neurodegeneration [75]. Furthermore, leptin could decrease tau phosphorylation, eliciting neuroprotective effects. Aggregation and hyperphosphorylation of tau contribute to cytotoxicity and the onset of neurodegenerative diseases. This heightened phosphorylation elevates the risk of developing dementia [76].

Mutations in the *POMC* gene have been linked to morbid obesity [67]. *POMC* plays a pivotal role in the melanocortin–leptin system. The association of *POMC* with neurodegenerative diseases has been established through research involving a specific AD animal model. The 3xTg-AD mouse model expresses three genes associated with dementia: *PSEN1 m 146 V*, *APPSwe*, and microtubule-associated protein tau (*tauP 301 L*). This model demonstrated the presence of plaque and tangle pathology, synaptic dysfunction, and exhibited βA and tau pathology. The gene expression in the hypothalamus revealed elevated mRNA levels of genes associated with inflammation and apoptosis, along with decreased expression of neurons producing *POMC* and *NPY* [77].

Recent neuroimaging findings indicate a decline in cortical thickness in overweight or obese individuals. Von Bank et al. [88] explain that under conditions of excessive energy availability, the adipose tissue stores lipids, and under fasting conditions, the adipose tissue releases triglycerides. Aging increases the presence of inflammatory cells in adipose tissue, causing adipocytes to undergo hyperplasia and hypertrophy. These aging-related changes in adipose tissue promote the development of metabolic diseases and AD.

The inflammation induced by obesity has the potential to impact various brain structures, including the hippocampus, cerebral cortex, brain stem, and amygdala [45]. Adipokines, originating from adipose tissue, are produced both peripherally and within the CNS. Adipokines produced in the peripheral regions can cross the BBB or modify its physiology by affecting the cells that make up the BBB, impacting the CNS [78].

Outside of the CNS, adipose tissue macrophages infiltrate the adipose tissue, which contributes to insulin resistance. “Classically activated” macrophages are stimulated by proinflammatory mediators like IFN-γ and lipopolysaccharides. These M1 macrophages enhance the production of proinflammatory cytokines (such as IL-6, IL-12, and TNF-α), which impede the insulin action on adipocytes, linking inflammation with insulin resistance. Moreover, they produce ROS, including nitric oxide [78].

In the CNS, both local and systemic inflammation triggered by obesity can lead to an escalation in immune cell infiltration, a decrease in waste removal, and failure of the BBB. Activated microglia release proinflammatory cytokines, such as IFN-γ, TNF-α, IL-1β, and IL-6 [79].

Recent studies have consistently demonstrated a gray matter volume decrease in obese individuals, particularly in control regions [80]. Gomez et al. [78] revealed a positive correlation between BMI and the volume of specific regions, include the cingulum, corticospinal tract, internal capsule, uncinate fascicle, corpus callosum, inferior and superior longitudinal fascicles, inferior fronto-occipital fascicle, and the anterior and posterior thalamic radiations [78]. Among the obese population, there is an enlargement of the globus pallidus, putamen, and bilateral thalamus, while the bilateral caudate is diminished compared with the normal-weight subjects [78]. Notably, there is a significant reduction in the bilateral caudate medial–dorsal part, which is accompanied by a substantial increase in the bilateral thalamus lateral dorsal part in obese subjects [78].

Another aspect to be considered is mitochondrial dysfunction.

Growing evidence indicates that a high-fat diet can impair mitochondrial function, diminishing its oxidative capacity in both the brain cortex and the synaptosomal fraction [89]. 

This leads to increases in lipid peroxidation, ROS production, and cytochrome c oxidase activity, while resulting in a decrease in fatty acid oxidation and ATP production; these result in an overall decline in brain function, which is associated with cognitive impairment [45].

Another new aspect pertains to the gut–brain axis.

An impaired intestinal barrier, observed in high-fat diets and in obese subjects, can lead to the systemic dissemination of microbial metabolites and pathogens. This can compromise the BBB integrity and lead to neuroinflammation [90].

For example, free lipopolysaccharide (LPS) in the blood binds to the TLR4/CD14 complex in brain microglia, which then activates NF-κB and increases the production of cytokines like IL-1, IL-6, and TNF-α [45].

In AD, there is evidence that a gut-microbiome-mediated release of cytokines contributes to the formation of βA plaques and neurofibrillary tangles [91].

### 3.7. Limitations

This study has some limitations. First, the search was limited to the last five years of the literature, potentially excluding previous studies that might offer historical perspectives or significant insights into the relationship between body composition and Alzheimer’s disease.

A further limitation of this work concerns the failure to explore the influence of lifestyle factors, such as changes in diet and physical activity, on both body composition and Alzheimer’s disease risk. These factors, known to have a significant impact on public health, could offer new insights into Alzheimer’s disease prevention and targeted interventions with regard to body composition. Therefore, further studies are planned that will more thoroughly examine the influence of lifestyle modifications on AD progression and development.

## 4. Materials and Methods

The literature review was conducted from January to August 2024 using scientific databases, including PubMed, Google Scholar, and Cochrane Library. The focus was on identifying reviews related to AD and body composition. The filters used were “free full text”, “review”, and “5 years”.

Specific keywords were employed to refine the search: “body composition AND Alzheimer’s Disease”, “lean mass AND Alzheimer’s Disease”, “bone mass AND Alzheimer’s Disease”, and “fat mass AND Alzheimer’s Disease”. Only reviews published in peer-reviewed journals that explicitly mentioned body composition and AD in their title, abstract, or text were considered for inclusion.

The search process was carried out independently by three operators following the Preferred Reporting Items for Systematic Reviews and Meta-Analyses (PRISMA) guidelines [92] and the REAPPRAISED checklist. The selection procedure began with an initial examination of the titles, followed by the abstracts, and subsequently the full texts. Duplicate articles were identified and removed after a thorough review of the titles.

The selection process involved 167 articles. Fourteen duplicate articles were removed, and, in total, 146 articles were initially identified. After a preliminary assessment of the titles and abstracts, 56 articles were excluded. Subsequently, after full-text screening, 34 more articles were excluded. Ultimately, 56 articles meeting the criteria and relevant to the topic were included, comprising 51 reviews, 1 meta-analysis, 3 systematic reviews and meta-analyses, and 1 mini-review.

**Figure 1:**
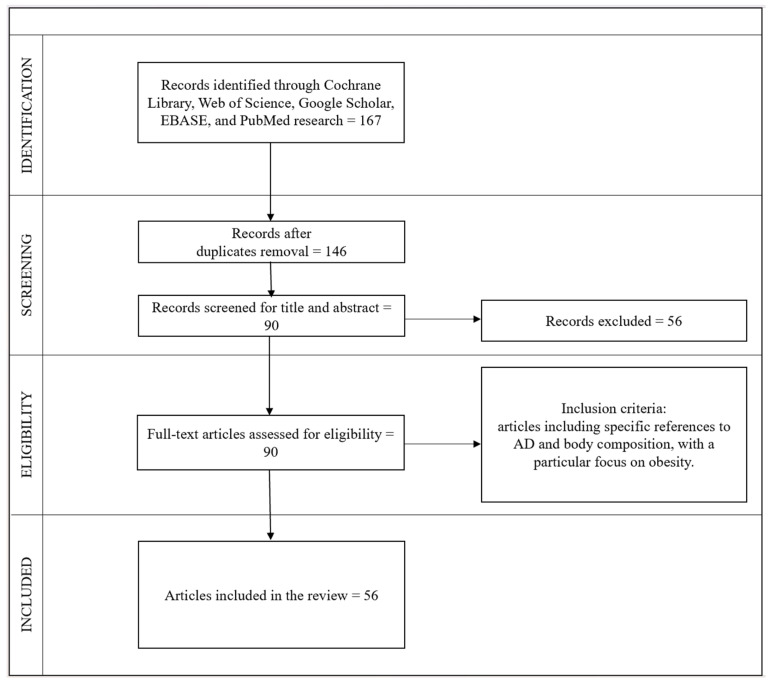
Flow diagram of the study selection.

Figure 1 illustrates the selection, inclusion, and analysis process.

## 5. Conclusions

This extensive analysis of the recent scientific literature underscores the multifaceted connections between AD and body composition. Lean mass, bone mass, and obesity-related factors, such as endocrinological imbalances and inflammation, have emerged as critical players in AD development. Molecular and genetic factors further contribute to the intricate web of relationships. The findings highlight the need for holistic approaches to address the AD risk, incorporating lifestyle modifications, immunomodulation, and targeted interventions. Enhanced comprehension of these connections could pave the way for innovative strategies in AD diagnosis, prevention, and treatment.

## Figures and Tables

**Table 1:** Summary of characteristics of the included studies on lean mass and AD in the most recent reviews, systematic reviews, and meta-analyses.

Authors	Title	Type of Article	Date	Key Findings
Wu, M.Y. et al. [25]	APP in the neuromuscular junction for the development of sarcopenia and Alzheimer’s disease.	Review	2023	APP, a key protein in AD development, is expressed in various tissues, including skeletal muscle, where its metabolism is crucial for NMJ synapse development and maintenance. The association of APP/βA pathology in muscles with myopathy in neurodegenerative disorders, such as AD and ALS, suggests a connection between muscular APP and brain pathology, shedding light on muscle–brain crosstalk and potential contributions to age-related degeneration.
Correa-de-Araujo, R. et al. [26]	Myosteatosis in the context of skeletal muscle function deficit: an interdisciplinary workshop at the National Institute on Aging.	Review	2020	The role of myosteatosis in muscle aging and metabolic diseases is highlighted, and determinants, consequences, and methods of evaluation are explored. Innovative topics, such as the impact of circadian rhythms on skeletal muscle and the relationship with myosteatosis, are addressed. The muscle–bone interaction perspective highlights preventive approaches, such as physical activity, myostatin treatment, and caloric restriction. The impact of myosteatosis on cancer survivors opens up new perspectives for identifying its role through interdisciplinary collaborations.
Ingenbleek, Y. et al. [27]	Implications of protein malnutrition and inflammatory disorders in the pathophysiology of Alzheimer’s disease.	Review	2020	Inflammatory states and malnutrition result in a reduced LBM and, consequently, in reduced nitrogen stores. Nitrogen and sulfur depletion from LBM stores could contribute to brain deterioration.
Suryadevara, V. et al. [28]	The unraveling: cardiac and musculoskeletal defects and their role in common Alzheimer disease morbidity and mortality.	Review	2020	Skeletal muscle dysfunction could precede the onset of AD-related symptoms and serve as a predictor of cognitive decline. Sarcopenia and dynapenia correlate with the severity of AD. Unsteady gait is considered a prodromal sign of cognitive decline.
Halon-Golabek, M. et al. [29]	Iron metabolism of the skeletal muscle and neurodegeneration.	Review	2019	Skeletal muscle has endocrine functions essential for a long and healthy life. Iron accumulation in muscle increases oxidative stress and is neurotoxic. Exercise stimulates the release of myokines, aids skeletal muscle wellness, reduces skeletal muscle iron stores, and consequently lowers the risk of AD.
Han, X. et al. [30]	Muscle–brain crosstalk in cognitive impairment.	Review	2023	The authors suggest a strong association between sarcopenia, cognitive impairment, and AD. They identify factors that maintain muscle trophism and that are neuroprotective, including IGF-1, BDNF, irisin, and SPARC. Aerobic and resistance exercise maintains muscle mass and would appear to have positive effects in maintaining cognitive health.
Brisendine, M.H. et al. [31]	Early-stage Alzheimer’s disease: are skeletal muscle and exercise the key?	Review	2023	The presence of a close relationship between skeletal muscles and cognitive function is emphasized. Resistance exercise appears to preserve cognitive function and reduce AD-related morbidity.
García-Llorente, A.M. et al. [32]	Mutidomain interventions for sarcopenia and cognitive flexibility in older adults for promoting healthy aging: a systematic review and meta-analysis of randomized controlled trials.	Review and meta-analysis	2024	Cognitive and physical training conducted for at least 8 weeks is shown to be effective in improving muscle strength and cognitive flexibility in the elderly. Therefore, multidomain training can contribute to the prevention and treatment of age-related diseases, including AD.
Jodeiri Farshbaf, M. et al. [33]	Multiple roles in neuroprotection for the exercise-derived myokine irisin.	Review	2021	*FNDC5*/Irisin expression has a neuroprotective role against the development of AD, as it reduces the formation of βA deposits, neuronal apoptosis, and neuroinflammation. Its expression is increased during exercise. Irisin administration results in a reduction in depressive and anxiety symptoms and memory impairment.
Chen, K. et al. [34]	Protective effect of irisin against Alzheimer’s disease.	Review	2022	Irisin has a protective role against AD through several mechanisms. It promotes learning and memory. Mechanisms that reduce the risk of AD include a reduction in the levels of proinflammatory cytokines (IL-6 and IL-1β) and a reduction in insulin resistance.
Cao, X. et al. [35]	Exercise drives metabolic integration between muscle, adipose and liver metabolism and protects against aging-related diseases.	Review	2023	Exercise reduces the risk of AD through several mechanisms that are not fully elucidated. Irisin would appear to have a protective and therapeutic role in AD; however, further research is needed to clarify its use in clinical practice.

Abbreviations: AD, Alzheimer’s disease; ALS, amyotrophic lateral sclerosis; APP, amyloid protein precursor; βA, β-amyloid; BDNF, brain-derived neurotrophic factor; IL, interleukin; IGF, insulin-growth factor; LBM, lean body mass; NMJ, neuromuscular junction; SPARC, secreted protein acidic and rich in cysteine.

**Table 2:** Summary of characteristics of the included studies on bone mass and AD in the most recent reviews, systematic reviews, and meta-analyses.

Authors	Title	Type of Article	Date	Key Findings
Karnik, S.J. et al. [36]	Mind the gap: unraveling the intricate dance between Alzheimer’s disease and related dementias and bone health.	Review	2024	AD and bone pathology have a bidirectional cause–effect relationship. The neuroinflammation present in AD seems to cause dysregulation of the hypothalamic–pituitary–adrenal axis with a consequent increase in cortisol, which subsequently leads to a loss of bone density. Conversely, chronic inflammation, resulting from osteoporosis and fractures, contributes to the pathogenesis of AD. AD patients have an increased risk of fractures due to the increased risk of falls and the reduced BMD. On the other hand, although it seems that fractures might promote the progression of AD, there is currently insufficient evidence to support this
Frame, G. et al. [37]	Mechanistic complexities of bone loss in Alzheimer’s disease: a review.	Review	2020	Bone loss in AD occurs early and is not associated with aging, sex, mobility, or genetics. Despite preliminary research, possible disruptive mechanisms of skeletal homeostasis have emerged, including the effects of amyloid-beta on bone cells and the damage caused by neurofibrillary tau in neural centers that regulate skeletal remodeling, along with deficits in systemic Wnt/β-catenin signaling. Irisin and *FNDC5* have implicated roles in AD-related bone loss and neurodegeneration. The current research is considered insufficient, requiring critical attention for possible new diagnostic and therapeutic opportunities.
Zhou, B.N. et al. [38]	Alzheimer’s disease and its associated risk of bone fractures: a narrative review.	Review	2023	AD patients have a significant risk of bone fractures, which are attributable to multiple factors, such as the direct effects of amyloid pathology on bone cells, an abnormal connection between brain and bone, deficits in Wnt/β-catenin signaling, reduced activity, high risk of falls, frailty, and chronic immune activity. Exercise, fall prevention, and an enriched diet are found to be beneficial in reducing the fracture risk. However, the efficacy of anti-osteoporotic agents needs to be further evaluated in AD patients, as clinical trials are limited.
Ruggiero, C. et al. [39]	Dementia, osteoporosis and fragility fractures: intricate epidemiological relationships, plausible biological connections, and twisted clinical practices.	Review	2023	AD patients have twice the risk of fragility fractures compared with healthy people. Individuals aged >65 years have a 60% risk of dementia following hip fractures, a 47% risk following vertebral fractures, and a 35% risk following lower limb fractures. Several molecular mechanisms would appear to be common to osteoporosis and cognitive impairment. The pathophysiological pathways underlying the complex interaction between bone and brain involve neuropeptides, osteokines, and hormones. Some of these molecules, including NPY, vitamin D, OCN, leptin and glutamate, are being investigated. However, understanding their interactions in the context of changes in body composition and dietary habits remains a topic of study. The gut microbiota is being studied to create innovative interventions to improve the well-being of older individuals at risk of these interconnected health challenges.
Zhao, Y. et al. [40]	Cognitive impairment and risks of osteoporosis: a systematic review and meta-analysis.	Systematic review/ meta-analysis	2022	AD patients have a worse bone status than healthy people. Patients with osteoporosis have an increased risk of cognitive impairment (OR = 2.01, 95% CI: 1.63-2.48) and the same is true for patients with reduced BMD. Osteoporosis treatment could prevent or delay the onset of cognitive impairment. This article analyzes possible interactions between osteoporosis and cognitive impairment. Specifically, AD is associated with βA deposition, and it is highlighted that βA can increase *RANKL* activation, and thus osteoclast activation, by increasing bone resorption. In addition, low levels of osteocalcin, a marker of bone formation, reflect both the severity of osteoporosis and the severity of cognitive impairment.

Abbreviations: AD, Alzheimer’s dDisease; βA, β-amyloid; BMD, bone mass density; FNDC5, fibronectin type III domain-containing protein 5; NPY, neuropeptide Y; OCN, osteocalcin; RANKL, receptor activator of nuclear factor-kb ligand.

**Table 3:** Summary of characteristics of the included studies on the relationship between fat mass and AD from an endocrinological perspective in the most recent reviews, systematic reviews, and meta-analyses.

Authors	Title	Type of Article	Date	Key Findings
Sindzingre, L. et al. [41]	The role of adiponectin in Alzheimer’s disease: a translational review.	Review	2024	While preclinical studies have shown that adiponectin has a neuroprotective role in preventing AD, human studies have shown conflicting results. Therefore, the non-unique results in humans support the need for further research.
Casado, M.E. et al. [42]	Recent advances in the knowledge of the mechanisms of leptin physiology and actions in neurological and metabolic pathologies.	Review	2023	Excess weight correlates with low-grade inflammation due to the production of adipokines. These include leptin, the secretion of which is directly proportional to the amount of adipose tissue. Leptin is neuroprotective. Therefore, central leptin resistance, which is established in obese individuals, correlates with some neurological diseases, including AD.
Andrade, L.J. et al. [43]	Brain insulin resistance and Alzheimer’s disease: a systematic review.	Review	2023	Brain insulin resistance is present in several neurodegenerative diseases. Some studies show that impaired brain glucose utilization correlates with reduced neuronal plasticity and cognitive impairment; however, some research studies do not support a relationship between brain insulin resistance and AD. Therefore, further studies are needed to determine whether or not there is a causal relationship between insulin resistance and AD.
Huber, K. et al. [44]	The role of adipokines in the pathologies of the central nervous system.	Review	2023	The role of thirteen adipokines in the pathologies of the central nervous system are analyzed. In particular, cystatin C, adiponectin, and leptin have a neuroprotective role so they counteract AD development. Chitinase 3-like protein 1 promotes proinflammatory responses and is elevated in the early stages of AD.
Neto, A. et al. [45]	The complex relationship between obesity and neurodegenerative diseases: an updated review.	Review	2023	Obesity is associated with low-grade inflammation and is a risk factor for AD development and progression. This review analyzes the mechanisms involved in the interaction between obesity and AD, such as leptin resistance, low levels of adiponectin, insulin resistance, oxidative stress, βA disturbances, and brain atrophy.
Abdalla, M.M.I. [46]	Insulin resistance as the molecular link between diabetes and Alzheimer’s disease.	Review	2024	Increased AD risk has been linked to diabetes mellitus, with insulin resistance playing a key role in AD development. Dysfunctional insulin signaling in the brain may contribute to hallmark AD features like βA plaques and tau protein tangles, suggesting that targeting insulin resistance could offer new treatment possibilities for AD.
Arjunan, A. et al. [47]	Identification of the molecular mechanism of insulin-like growth factor-1 (IGF-1): a promising therapeutic target for neurodegenerative diseases associated with metabolic syndrome.	Review	2023	Recent evidence links metabolic syndrome to neurodegenerative diseases like AD, with factors such as hyperglycemia and abdominal obesity contributing to disease progression. IGF-1 deficiency, associated with metabolic syndrome-related pathologies, may play a key role in AD, highlighting IGF-1 as a potential therapeutic target for treating metabolic-syndrome-related neurodegeneration.
Tasnim, N. et al. [48]	Exploring the effects of adiponectin and leptin in correlating obesity with cognitive decline: a systematic review.	Systematic review/meta-analysis	2023	Obese patients frequently suffer from cognitive decline, dementia, and AD. This review analyzes the neurological effects of adiponectin and leptin in obese patients. Adiponectin and leptin are the two major adipokines that have a preventive role against obesity and dementia, and both are involved in the causal relationship between obesity and cognitive decline.
Cimini, F.A. et al. [49]	Role of biliverdin reductase A in the regulation of insulin signaling in metabolic and neurodegenerative diseases: an update.	Review	2022	Insulin resistance is a feature of obesity, metabolic syndrome, and type 2 diabetes, and it is linked with cognitive impairment. Among the mechanisms involved in the development of insulin resistance is that of BVR-A, which acts as a regulator of insulin signaling.
Flores-Cordero, J.A. et al. [50]	Obesity as a risk factor for dementia and Alzheimer’s disease: the role of leptin.	Review	2022	In obesity, chronic low-grade inflammation is responsible for leptin resistance, which, in turn, correlates with the development of AD.
Chung, K.W. et al. [51]	Advances in understanding of the role of lipid metabolism in aging.	Review	2021	During aging, the amount of adipose tissue increases, and the formation of ectopic fat occurs. This process is responsible for lipotoxicity, reduced energy availability, and altered cellular signaling mechanisms. These factors contribute to the development of AD.
Khoramipour, K. et al. [52]	Adiponectin: structure, physiological functions, role in diseases, and effects of nutrition.	Review	2021	Adiponectin regulates energy homeostasis, promotes hippocampal neurogenesis and neuronal plasticity, and has positive effects on cognitive function. Its secretion increases after a healthy diet and is reduced in obese subjects. Reduction of adiponectin promotes the progression of AD and causes cognitive impairment.
Komleva, Y. et al. [53]	Inflamm-aging and brain insulin resistance: new insights and role of life-style strategies on cognitive and social determinants in aging and neurodegeneration.	Review	2021	AD is associated with aging and is characterized by inflammation; otherwise known as inflammaging. This review analyzes the role of nutrients, obesity, low-grade chronic inflammation linked with obesity in brain aging, neurodegeneration, cognitive decline, and AD. Obesity causes inflammation and insulin resistance, which, in turn, promotes the development of AD.
Polito, R. et al. [54]	Adiponectin role in neurodegenerative diseases: focus on nutrition.	Review	2020	Adiponectin has a role in the prevention of neurodegenerative diseases such as AD. A reduction of adiponectin correlates with neurodegenerative disease severity, and this is observed in obese patients, suggesting a correlation between AD and obesity.
Kim, J.Y. et al. [55]	Adiponectin: the potential regulator and therapeutic target of obesity and Alzheimer’s disease.	Review	2020	There is an association between obesity, type 2 diabetes, and AD. Adiponectin increases insulin sensitivity, reduces inflammation, and is neuroprotective. Adiponectin levels are inversely proportional to the amount of central adipose tissue. Reduced levels of adiponectin are found in mild cognitive impairment and AD.
Forny-Germano, L. et al. [56]	The Role of leptin and adiponectin in obesity-associated cognitive decline and Alzheimer’s disease.	Review	2019	Obesity has been linked to an increased risk of AD due to dysregulated adipokines like leptin and adiponectin, which are critical in brain function. Dysfunctions in these adipokines may contribute to AD-related neuropathological events, including amyloid buildup, tau hyperphosphorylation, and neuroinflammation, suggesting that restoring their proper signaling could be a potential therapeutic strategy for AD.
Uddin, M.S. et al. [57]	Exploring the new horizon of adipoQ in obesity-related Alzheimer’s dementia.	Review	2021	Obesity is a risk factor for the development and progression of AD. Adiponectin has neuroprotective activity, reduces βA deposit formation, neuroinflammation, and insulin resistance; and increases neuronal plasticity. In obese patients, adiponectin is reduced, and this correlates with an increased risk of developing AD.
Huang, X. et al. [58]	Bidirectional communication between brain and visceral white adipose tissue: its potential impact on Alzheimer’s disease.	Review	2022	This review highlights that visceral white adipose tissue contributes to the development and progression of AD and analyzes the mechanisms involved.
Farruggia, M.C. et al. [59]	Effects of adiposity and metabolic dysfunction on cognition: a review.	Review	2019	Adiposity and metabolic dysfunction, such as insulin resistance and type 2 diabetes, are independent risk factors for dementia and AD in animal models, whereas in humans, it remains uncertain whether these risk factors are independent.

Abbreviations: AD, Alzheimer’s disease; βA, β-amyloid; BVR-A, biliverdin reductase-A, IGF-1, insulin growth factor-1.

**Table 4:** Summary of characteristics of the included studies on the relationship between fat mass and AD from an immunological perspective in the most recent reviews, systematic reviews, and meta-analyses.

Authors	Title	Type of Article	Date	Key Findings
Kueck, P.J. et al. [60]	Current perspectives: obesity and neurodegeneration—links and risks.	Review	2023	Obesity-induced metabolic changes, including insulin resistance and increased oxidative stress, disrupt energy expenditure and mitochondrial function, contributing to cognitive decline and an increased risk of AD. The resulting oxidative stress damages lipids, proteins, and DNA, leading to endothelial dysfunction and reduced white matter integrity, exacerbating neurodegeneration in AD.
Woo, A. et al. [61]	Obesity-related neuroinflammation: magnetic resonance and microscopy imaging of the brain.	Mini-review	2022	Higher adiposity, especially visceral fat, is associated with structural brain changes such as reduced cortical thickness and increased white matter hyperintensities, contributing to neuroinflammation and potentially impairing cognitive functions related to AD. Microscopy imaging reveals that obesity-related cellular changes, like reduced dendritic spine density and increased activation of microglia and astrocytes, disrupt the blood–brain barrier and promote neuroinflammation, further contributing to AD.
Litwiniuk, A. et al. [62]	Inflammasome NLRP3 potentially links obesity-associated low-grade systemic inflammation and insulin resistance with Alzheimer’s disease.	Review	2021	AD involves a progressive decline in memory and cognitive functions that is linked to βA accumulation, tau phosphorylation, mitochondrial damage, synaptic loss, and neuroinflammation. The activation of inflammasomes like NLRP3 exacerbates neuroinflammation and contributes to AD pathology, suggesting potential therapeutic avenues targeting these pathways to mitigate cognitive decline.
Pichiah, P.B.T. et al. [63]	Derived molecules—untouched horizons in Alzheimer’s disease biology.	Review	2020	Adipose-tissue-derived cytokines and proteins, including IL-1β, IL-4, IL-10, IL-18, TNFα, MIF, CRP, chemerin, RANTES, PAI-1, CFH, adiponectin, CETP, LPL, RBP4, and resistin, contribute to AD progression by influencing neuroinflammation, βA accumulation, and potentially affecting autophagy pathways. Their roles underscore the complex interplay between obesity-related factors and AD pathophysiology, suggesting potential therapeutic targets for managing the disease.
Kang, J.W. et al. [64]	The potential utility of prebiotics to modulate Alzheimer’s disease: a review of the evidence.	Review	2021	Evidence suggests that promoting beneficial microbes, particularly SCFA-producing ones like *Bifidobacteria*, through prebiotic interventions has the potential to alleviate AD-associated symptoms. Different types of prebiotics, especially fructans, have shown efficacy in modulating gut microbiome composition and metabolite production.
Lu, Y. et al. [65]	Advances in the study of IL-17 in neurological diseases and mental disorders.	Review	2023	IL-17 is increasingly recognized as a crucial mediator of immune regulation through neuroinflammation and the microbiota–gut–brain axis. The complex interplay of factors influencing the human neurological system and gut microbiota, along with ongoing debates about the translatability of animal models to humans, warrants further investigation into the role of IL-17 in disease pathogenesis and the potential for targeted therapeutic interventions.
Natale, G. et al. [66]	Obesity in late-life as a protective factor against dementia and dementia-related mortality.	Review	2023	A protective effect of later-life obesity against cognitive decline, dementia, and dementia-related mortality is suggested, challenging the notion that obesity is a direct risk factor for these conditions. The findings emphasize the need for further exploration of the complex relationships between obesity, age, and cardiometabolic risks, especially considering the increasing prevalence of obesity in younger populations and its potential implications for future dementia incidence.
Flores-Dorantes, M.T. et al. [67]	Environment and gene association with obesity and their impact on neurodegenerative and neurodevelopmental diseases.	Review	2020	The article explores gene–environment interactions in obesity, focusing on genetic variants like *FTO*, *MC4R*, *LEP*, *LEPR*, *POMC*, *CART*, *NPY*, *PCSK1*, *SIM1*, *BDNF*, and *TrKB*, which influence obesity susceptibility through pathways affecting energy balance and neurodegenerative/neurodevelopmental diseases. It underscores the shared genetic mechanisms linking obesity with AD, Parkinson’s disease, and schizophrenia, suggesting potential therapeutic targets across these complex disorders.
Cianci, R. et al. [68]	The crosstalk between gut microbiota, intestinal immunological niche and visceral adipose tissue as a new model for the pathogenesis of metabolic and inflammatory diseases: the paradigm of type 2 diabetes mellitus.	Review	2022	The GM plays a crucial role in a wide range of molecular interactions, influencing endocrine functions, immune responses, and metabolism. The bidirectional interaction between GM and VAT is pivotal, impacting adipokines, hormones, and immune reactions and contributing to conditions such as diabetes and other metabolic disorders, suggesting potential therapeutic avenues for these conditions.
Kuneš, J. et al. [69]	Obesity, cardiovascular and neurodegenerative diseases: potential common mechanisms.	Review	2023	Obesity, CVD, and neurodegenerative disorders often co-occur and share mechanisms like inflammation and oxidative stress. Emphasizing lifestyle modifications, such as physical activity and a healthy diet, is crucial for prevention, considering the absence of effective drugs for obesity and neurodegenerative diseases, unlike available options for CVD, which may also influence cognitive decline and vascular dementia.
Al-Kuraishy, H.M. et al. [70]	A potential link between visceral obesity and risk of Alzheimer’s disease.	Review	2022	AD, featuring brain plaques and tangles, may have a connection to visceral obesity with inflammation and adipocytokine irregularities. Visceral obesity-induced oxidative stress and inflammation could contribute to AD development, emphasizing the importance of early recognition and management for potential prevention.
Ingenbleek, Y. et al. [71]	Plasma transthyretin as a biomarker of sarcopenia in elderly subjects.	Review	2019	Plasma TTR serves not only as a carrier protein for thyroid hormones and retinol but also exhibits neuroprotective properties when secreted by the brain choroid plexus. While TTR has been associated with amyloidogenic processes leading to morbidities in various tissues, recent data contest previous objections and establish that TTR is an active contributor in inflammatory responses, releasing free fractions of thyroxine and retinol in stressful disorders and strengthening the effects initiated by cytokines.
Ioannou, A. et al. [72]	Patisiran for the treatment of transthyretin-mediated amyloidosis with cardiomyopathy.	Review	2023	Reducing circulating TTR concentrations with patisiran has shown promising results, leading to improvements in various cardiac and neurological measures, as in ATTR-CM. The potential expansion of patisiran’s license to include ATTR-CM could provide an additional disease-modifying treatment for this aggressive cardiomyopathy, although the long-term effects of patisiran-induced TTR depletion still need to be determined.
Ingenbleek, Y. et al. [73]	Revisiting PINI scoring in light of recent biological advances.	Review	2023	The PINI scoring system involves a ratio between inflammatory biomarkers in the numerator and liver biomarkers assessing the protein nutritional status (TTR and albumin) in the denominator. TTR, which uniquely correlates with lean body mass from birth to old age, plays a crucial role in quantifying protein depletion during inflammatory disorders, making it a versatile indicator in various clinical conditions, including in neonates, critically ill patients, and those with conditions like AD.

Abbreviations: AD, Alzheimer’s disease; APOE, apolipoprotein E; ATTR-CM, transthyretin amyloid cardiomyopathy; βA, β-amyloid; BBB, blood–brain barrier; BDNF, brain-derived neurotrophic factor; CART, cocaine- and amphetamine-regulated transcript; CETP, cholesteryl ester transfer protein; CNS, central nervous system; CRP, C-reactive protein; CVD, cardiovascular disease; FTO, fat mass and obesity-associated gene; GM, gut microbiota; HMGCR, 3-hydroxy-3-methylglutaryl-CoA reductase; IL, interleukin; LEP, leptin; LEPR, leptin receptor; LPL, lipoprotein lipase; LPS, lipopolysaccharide; MC4R, melanocortin 4 receptor; MIF, macrophage migration inhibitory factor; NLRP3, NOD-, LRR- and pyrin-domain-containing protein 3; NPY, neuropeptide Y; NSAIDs, non-steroidal anti-inflammatory drugs; PAI1, plasminogen activator inhibitor-1; PCSK1, proprotein convertase subtilisin/kexin Type 1; PINI, prognostic inflammatory and nutritional index; POMC, proopiomelanocortin; RANTES, regulated upon activation, normal T cell expressed and secreted; RBP4, retinol-binding protein 4; SAE, sepsis-associated encephalopathy; SIM1, single-minded 1; TCR, T cell receptor; TrkB, tropomyosin receptor kinase B; SCFA, short-chain fatty acid; Th17, T helper 17 cells; TNFα, tumor necrosis factor α; Tregs, regulatory T cells; TTR, transthyretin; VAT, visceral adipose tissue.

**Table 5:** Summary of characteristics of the included studies on the relationship between fat mass and AD from a molecular and genetic perspective in the most recent reviews, systematic reviews, and meta-analyses.

Authors	Title	Type of Article	Date	Key Findings
Obradovic, M. et al. [74]	Leptin and obesity: role and clinical implication.	Review	2021	The discovery of leptin has shed light on obesity control, and alterations in its expression and receptor contribute to leptin resistance, a crucial factor in obesity-related complications. Despite its role as a principal appetite suppressor, utilizing leptin-based therapeutics for obesity treatment requires further exploration, emphasizing the need to identify new mechanisms of leptin regulation to design drugs that can reverse leptin resistance and offer alternatives in obesity treatment.
Sarapultsev, A. et al. [75]	JAK-STAT signaling in inflammation and stress-related diseases: implications for therapeutic interventions.	Review	2023	The multifaceted role of the JAK-STAT signaling pathway in systemic homeostasis, including its involvement in inflammation, meta-inflammation, and aging, prompts an exploration of its relevance to stress-associated disorders. Isoform-specific inhibitors like filgotinib and upadacitinib, along with combination therapies and precision drug delivery systems, offer a new era of targeted pharmacology that enriches therapeutic approaches and deepen our understanding of the pathway’s involvement in the complex interplay between stress and inflammation.
Amontree, M. et al. [76]	Matrix disequilibrium in Alzheimer’s disease and conditions that increase Alzheimer’s disease risk.	Review	2023	Common risk factors for late-onset AD, including age, *APOE* genotype, untreated major depressive disorder, and obesity, are associated with chronic inflammation and increased ECM deposition. The review suggests that excess ECM deposition may restrict neuroplasticity, impair cognitive reserves, and alter the excitatory/inhibitory balance, highlighting the importance of studying ECM interventions in AD and related risk factor models.
Reyna, N.C. et al. [77]	Anxiety and Alzheimer’s disease pathogenesis: focus on 5-HT and CRF systems in 3xTg-AD and TgF344-AD animal models.	Review	2023	Despite behavioral inconsistencies, both mouse models consistently exhibit robust AD pathology, with the TgF344-AD rat model showing more reliable behavioral findings and expressing neuroinflammation and neuronal loss, aspects lacking in mouse AD models. Targeting the 5-HT and CRF systems emerges as a promising strategy, with the 5-HT system offering a feasible preventative measure and CRF1 antagonists presenting a potential avenue to refine interventions and improve clinical outcomes in AD patients.
Gómez-Apo, E. et al. [78]	Structural brain changes associated with overweight and obesity.	Review	2021	Excessive visceral fat induces chronic inflammation affecting various tissues, including the brain, leading to structural abnormalities and reduced gray matter volume. Obesity is linked to neurodegenerative diseases, influencing synaptic plasticity, cognitive deficits, and behavioral patterns, while physiological changes such as chronic inflammation, insulin resistance, and microangiopathy contribute to neuronal loss and lower cortical thickness, explaining the poor cognitive performance in individuals with overweight and obesity.
Schmitt, L.O. et al. [79]	Obesity-induced brain neuroinflammatory and mitochondrial changes.	Review	2023	A high-fat and high-caloric diet intake, along with obesity and T2DM, induces brain dysfunction that is characterized by neuroinflammation and mitochondrial dysfunction. These mechanisms are proposed to contribute to cognitive deficits and dementia associated with metabolic disorders, and interventions targeting brain mitochondrial dysfunction and reducing oxidative stress levels in the hippocampus may have beneficial effects on cognitive processes.
Herrmann, M.J. et al. [80]	Grey matter alterations in obesity: a meta-analysis of whole-brain studies.	Meta-analysis	2019	Notable structural alterations in the GMV within the insula and cerebellum among individuals with obesity are identified that are associated with psychopathological symptoms. However, additional investigations are necessary to ascertain whether these GMV changes play a causal role, result from obesity-related factors, or simply co-occur with the condition.

Abbreviations: 5-HT, serotonin; AD, Alzheimer’s disease; APOE, apolipoprotein E; CRF, corticotropin-releasing factor; ECM, extracellular matrix; GMV, gray matter volume; T2DM, type 2 diabetes mellitus.

## Data Availability

Not applicable.
